# Oxidative Stress Mediates Anxiety-Like Behavior Induced by High Caffeine Intake in Zebrafish: Protective Effect of Alpha-Tocopherol

**DOI:** 10.1155/2019/8419810

**Published:** 2019-10-21

**Authors:** Tayana Silva de Carvalho, Patrick Bruno Cardoso, Mateus Santos-Silva, Sávio Lima-Bastos, Waldo Lucas Luz, Nadyme Assad, Nayara Kauffmann, Adelaide Passos, Alódia Brasil, Carlomagno Pacheco Bahia, Suellen Moraes, Amauri Gouveia, Evander de Jesus Oliveira Batista, Karen Renata Matos Herculano Oliveira, Anderson Manoel Herculano

**Affiliations:** ^1^Instituto de Ciências Biológicas, Universidade Federal do Pará, Belém, Pará, Brazil; ^2^Instituto de Ciências da Saúde, Universidade Federal do Pará, Belém, Pará, Brazil; ^3^Núcleo de Teoria e Pesquisa do Comportamento, Universidade Federal do Pará, Belém, Pará, Brazil; ^4^Núcleo de Medicina Tropical, Universidade Federal do Pará, Belém, Pará, Brazil

## Abstract

Anxiety is a common symptom associated with high caffeine intake. Although the neurochemical mechanisms of caffeine-induced anxiety remain unclear, there are some evidences suggesting participation of oxidative stress. Based on these evidences, the current study is aimed at evaluating the possible protective effect of alpha-tocopherol (TPH) against anxiety-like behavior induced by caffeine (CAF) in zebrafish. Adult animals were treated with CAF (100 mg/kg) or TPH (1 mg/kg)+CAF before behavioral and biochemical evaluations. Oxidative stress in the zebrafish brain was evaluated by a lipid peroxidation assay, and anxiety-like behavior was monitored using light/dark preference and novel tank diving test. Caffeine treatment evoked significant elevation of brain MDA levels in the zebrafish brain, and TPH treatment prevented this increase. Caffeine treatment also induced anxiety-like behavior, while this effect was not observed in the TPH+CAF group. Taken together, the current study suggests that TPH treatment is able to inhibit oxidative stress and anxiety-like behavior evoked by caffeine.

## 1. Introduction

Caffeine (1,3,7-trimethylxanthine) is a psychoactive drug widely consumed around the world [1]. The average of caffeine intake in developing countries is about 50 mg per day. However, this consumption can reach up to 400 mg per day in developed countries, such as Sweden, United States, and United Kingdom [2]. It is widely described that low doses of caffeine exerts a positive effect on cognition, memory, and learning [3], while high doses induce mental impairment, hyperactivity, and anxiety [4–6]. These finds suggest that caffeine is able to promote a dual effect on the central nervous system (CNS).

The broad effects of caffeine in CNS occur via nonspecific inhibition of adenosine receptors expressed either in neurons or in glial cells [7]. As previously described, low doses of caffeine antagonize A2a adenosine receptors, promoting motor changes and anxiolytic-like behavior responses [8]. On the other hand, it was also demonstrated that high doses of caffeine may induce anxiogenic-like behavior by unclear mechanisms [9]. In addition, the effect of caffeine on other neurotransmitter systems has been previously described in the literature. It is well described that caffeine is able to modulate the extracellular levels of glutamate as well as to prevent oxidative stress in the brain [10]. Although it is widely showed that glutamate and reactive oxygen species (ROS) lead to neurobehavioral alterations [11], it remains unclear if brain oxidative stress represents a biochemical mechanism involved in anxiety-like behavior evoked by caffeine.

Alpha-tocopherol is a chemical form of vitamin E which has potent antioxidant activity [12]. As confirmed in previous studies, alpha-tocopherol possess high liposolubility and easily crosses the blood-brain barrier [13]. This chemical property assures its effect on the CNS, even after a systemic administration. In case of alpha-tocopherol deficiency, increased anxiety-like behavior was previously reported in experimental studies [14]. Based on these evidences, it is reasonable to hypothesize that alpha-tocopherol can exert a neuroprotective role against the deleterious effects of caffeine on the CNS.

Zebrafish has emerged as a powerful animal model used in different neuropsychopharmacological studies [15]. It was recently demonstrated that caffeine induces anxiety-like behavior and autonomic arousal in zebrafish [16–18]. The existence of brain regions controlling anxiety-like behavior in zebrafish as well as its responsiveness to anxiolytic drugs validates the use of this animal model for preclinical evaluations of new anxiolytic agents [16]. Based on these findings, the current study is aimed at demonstrating that alpha-tocopherol treatment prevents anxiety-like behavior induced by caffeine in zebrafish.

## 2. Materials and Methods

### 2.1. Animals

166 longfin wild-type adult zebrafish were used in all experiments. Animals were acclimated in tank water held pH 6.0 at 28°C, with food dispensed once per day at light photoperiod controlled (12 : 12 light/dark). After 1 week of acclimation, the animals were submitted to experimental procedures.

### 2.2. Experimental Design and Drug Treatment

Zebrafish were distributed in four different groups as following: control group (CTRL), *α*-tocopherol group (TPH), caffeine group (CAF), and TPH+CAF group. The protocol of caffeine-induced anxiety was performed by zebrafish treatment with caffeine at 100 mg/kg as previously described by Maximino et al. [17]. In addition, a dose-response experiment was made in order to determinate the dose of alpha-tocopherol used in the current study. Alpha-tocopherol was diluted in 0.1% dimethyl sulfoxide (DMSO), and animals were treated with 1 mg/kg, 2 mg/kg, or 5 mg/kg as following (Supplementary [Supplementary-material supplementary-material-1]). Zebrafish were anesthetized in cold water at 2^o^C for 10 seconds being this step followed by intraperitoneal injection of 5 *μ*L of 0.9% saline solution (CTRL), 100 mg/kg caffeine (CAF), 1 mg/kg alpha-tocopherol (TPH) or 100 mg/kg caffeine+1 mg/kg alpha-tocopherol (TPH+CAF). After that, all groups were submitted to 30 minutes of habituation which was followed by the behavioral tests.

### 2.3. Light/Dark Preference (Scototaxis)

Scototaxis evaluation in control and treated groups was performed as described previously by Maximino et al. [19]. Briefly, after the treatments, zebrafish were individually transferred to the central compartment (delimited by doors) of a black/white tank (15 cm × 10 cm × 45 cm, *h* × *d* × *l*) for the habituation period (3 minutes). Afterwards, the doors were removed and the animal was allowed to freely explore the apparatus for 15 minutes. The behavioral parameters evaluated were time in the white compartment, thigmotaxis, erratic swimming, freezing, risk assessment, entries in the white compartment, latency to white, and squares crossed. Zebrafish behavior in the tank was recorded using a digital camera (Cyber-shot DSC-W710 BR4), and the videos were evaluated using X-Plo-Rat 1.1.0 software.

### 2.4. Novel Tank Diving Test (Geotaxis)

Control and treated animals were submitted to the novel tank diving test utilizing the modified protocols previously described by Egan et al. [20] and Cachat et al. [21]. After drug treatment, zebrafish were individually transferred to a glass aquarium (15 cm × 25 cm × 20 cm, width × length × height) and kept in the habituation period (3 minutes). After this time, the free exploration of the animal in the apparatus was recorded using a digital camera (Cyber-shot DSC-W710 BR4) for 6 minutes. The following parameters were analyzed: time on top, latency on top, squares crossed on top, erratic swimming, and freezing.

### 2.5. Lipid Peroxidation Assay

Animals were deeply anesthetized in cold solution as previously described by Maximino et al. [33]. Brains were mechanically dissociated and centrifuged at 5600 rpm for 10 minutes at 4°C. The product of lipid peroxidation was determined in supernatant utilizing pH 7.4 20 mM Tris-HCl containing 10.3 mM N-methyl-2-phenylindole (NMFI), methanosulfonic acid, acetonitrile, and methanol at 45^o^C. Analysis of the lipid peroxidation was carried out based on standard curve concentrations of malondialdehyde (MDA), measured by the absorbance at 570 nm wavelength. MDA concentration was quantified in nMols per milligram of protein, and the protein levels were determined by the Bradford method. The values were expressed as a percentage of the control.

### 2.6. Statistical Analysis

Data were expressed as the mean ± standard error. Normal distribution of the data was confirmed by the Shapiro-Wilk test, and the difference among the averages was evaluated using the ANOVA-one way test followed by the Tukey test. *p* < 0.05 was considered significant. All data were analyzed utilizing GraphPad Prism 5.0 software.

## 3. Results

### 3.1. Anxiety-Like Behavior Induced by Caffeine

**Figure 1:**
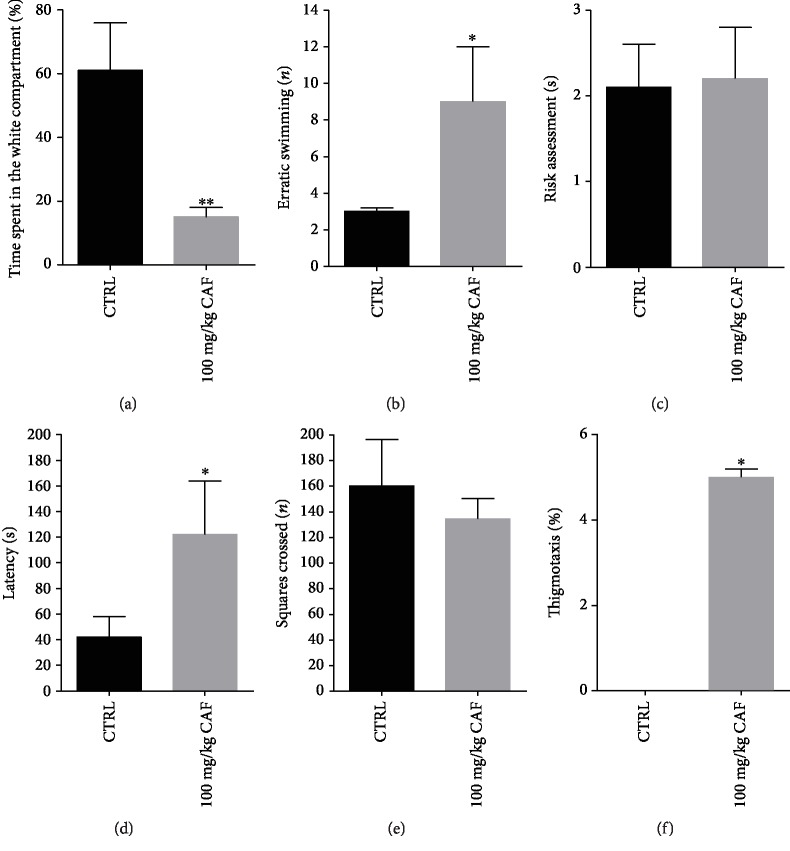
Effect of caffeine on (a) time spent in the white compartment, (b) erratic swimming, (c) risk assessment, (d) latency, (e) squares crossed, and (f) thigmotaxis in the scototaxis test. Bar graphs represent the mean ± standard error. Data were compared using the ANOVA-one way test followed by the Tukey test. ^∗^*p* < 0.05 vs. control.

Our results have shown that zebrafish treated with caffeine spent less time in the white compartment (Figure 1(a): CTRL = 62.24 ± 11.71%/CAF = 9.42 ± 2.50%; *F* = 19.5875; *p* < 0.01). We also evidenced significant behavior effects elicited by caffeine treatment in erratic swimming evaluation (Figure 1(b): CTRL = 2.43 ± 0.26/CAF = 9 ± 2.34; *F* = 6.8559, *p* < 0.05). Risk assessment was not altered in animals treated with caffeine (Figure 1(c): CTRL = 3 ± 0.61/CAF = 2.43 ± 0.44; *F* = 7.2864; *p* > 0.05). However, caffeine increased zebrafish latency on the white side of apparatus (Figure 1(d): CTRL = 70.14 ± 8.75 s/CAF = 145 ± 23.7 s; *p* < 0.05). Data of squares crossed results showed that caffeine did not alter locomotor activity in zebrafish (Figure 1(e): CTRL = 159.12 ± 37.43/CAF = 133.5 ± 17.95; *F* = 0.3333; *p* > 0.05). Our data also demonstrated that the caffeine-treated group had higher levels of wall hugging (thigmotaxis) when compared with the control (Figure 1(f): CTRL = 0/CAF = 4.8 ± 0.2).

**Figure 2:**
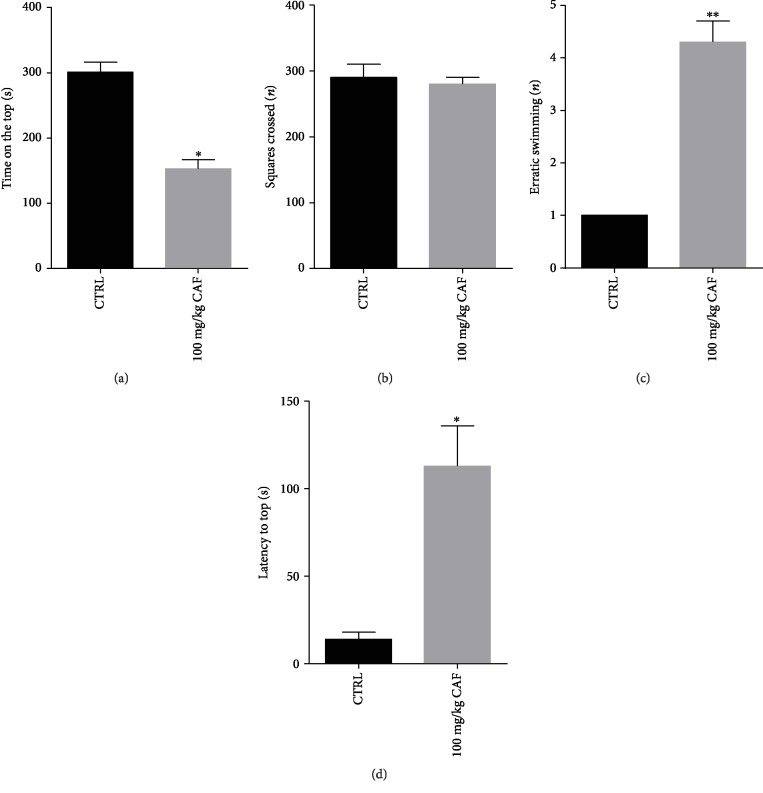
Effect of caffeine on (a) time on the top, (b) squares crossed, (c) erratic swimming, and (d) latency on the top in the geotaxy test. Bar graphs represent the mean ± standard error. Data were compared using the ANOVA-one way test followed by the Tukey test. ^∗∗^*p* < 0.05 vs. control and ^#^*p* < 0.05 vs. caffeine group.

Data regarding the novel tank test also showed anxiogenic-like effect of caffeine in zebrafish. Animals treated with caffeine spent low time on the top of the apparatus (Figure 2(a): CTRL = 311.53 ± 18.24 s/CAF = 151.5 ± 9.945 s; *F* = 7.9057; *p* < 0.05). As observed in the light/dark test, no locomotor alterations were evidenced in zebrafish treated with caffeine (Figure 2(b): CTRL = 295.5 ± 25.9/CAF = 274 ± 15.6; *F* = 0.3073; *p* > 0.05). Freezing values were higher in the caffeine group than the control group (data not shown). Values of erratic swimming also were significantly higher in animals treated with caffeine (Figure 2(c): CTRL = 1 vs. CAF = 11.6 ± 0.72; *F* = 38.5714; *p* < 0.01) as well as the latency to top (Figure 2(d): CTRL = 13.5 ± 3.75 s/CAF = 114 ± 30.9 s; *F* = 10.3957; *p* < 0.05).

### 3.2. Alpha-Tocopherol Effect on the Anxiety-Like Behavior

**Figure 3:**
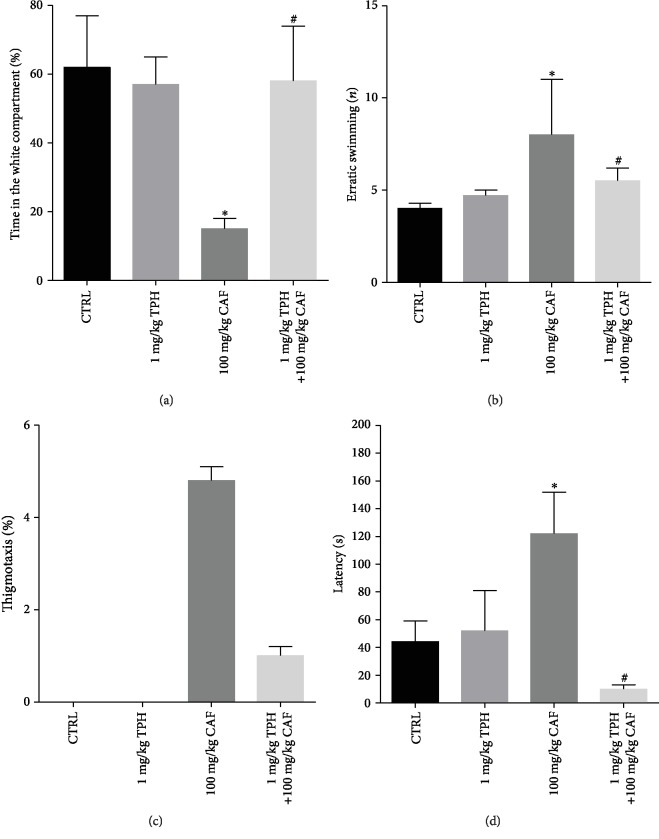
Effect of alpha-tocopherol on (a) time spent in the white compartment, (b) erratic swimming, (c) risk assessment, (d) latency, (e) squares crossed, and (f) thigmotaxis in the scototaxis of zebrafish treated with caffeine. Bar graphs represent the mean ± standard error. Data were compared using the ANOVA-one way test followed by the Tukey test. ^∗^*p* < 0.05 vs. control and ^#^*p* < 0.05 vs. caffeine group.

As observed in Supplementary [Supplementary-material supplementary-material-1], TPH at 2 mg/kg and 5 mg/kg evoked anxiolytic and anxiogenic-like behavior in zebrafish, respectively. However, alpha-tocopherol at 1 mg/kg did not evoke significant effect on the zebrafish anxiety-like behavior. Our data regarding the effect of TPH on the caffeine-induced anxiety showed that 1 mg/kg alpha-tocopherol blocked the decrease in the time spent in the white compartment evoked by caffeine treatment (Figure 3(a), CTRL = 62.24 ± 11.71%/CAF = 9.4 ± 2.9 vs. TPH + CAF = 52.04 ± 10.7; *F* = 4.944; *p* < 0.05). Similarly, 1 mg/kg alpha-tocopherol has partially prevented the erratic swimming observed in zebrafish treated with caffeine (Figure 3(b), CTRL = 2.43 ± 0.26/CAF = 9 ± 2.6 vs. TPH + CAF = 5.25 ± 0.64; *p* < 0.05). Alpha-tocopherol also exerted a significant effect on the caffeine-induced thigmotaxis (Figure 3(c), CAF = 8.41 ± 1.6 vs. TPH + CAF = 0.84 ± 0.10; *F* = 6.902; *p* < 0.05). The values of thigmotaxis were increased in the caffeine group when compared to the control group. These values were significantly decreased in animals cotreated with caffeine and alpha-tocopherol. We observed that latency to the white compartment was altered by alpha-tocopherol treatment when compared with the caffeine group (Figure 3(d), CTRL = 70.14 ± 8.75 s/CAF = 145 ± 23.7 s vs. TPH + CAF = 10 ± 3.7 s; *F* = 4 5.990; *p* < 0.05).

**Figure 4:**
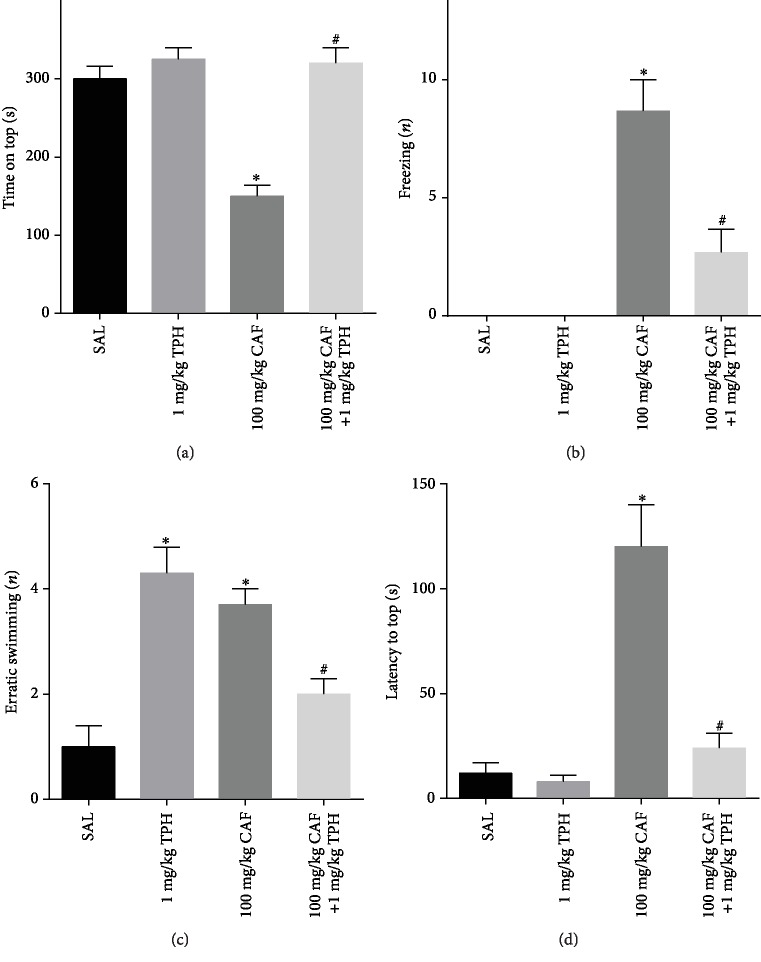
Effect of alpha-tocopherol on (a) time on the top, (b) squares crossed, (c) erratic swimming, and (d) latency on the top in the geotaxy of zebrafish treated with caffeine. Bar graphs represent the mean ± standard error. Data were compared using the ANOVA-one way test followed by the Tukey test. ^∗^*p* < 0.05 and ^∗∗^*p* < 0.05 vs. control and ^#^*p* < 0.05 vs. caffeine group.

Results of the novel tank diving test also have suggested anxiolytic-like behavior induced by alpha-tocopherol. Animals treated with caffeine decreased its time on the top of the aquarium, and this effect was significantly prevented by alpha-tocopherol (Figure 4(a), CTRL = 311.53 ± 18.24 s/CAF = 151.5 ± 9.945 s vs. TPH + CAF = 331.4 ± 19.6 s; *F* = 5.159; *p* < 0.05). Alpha-tocopherol treatment also has blocked freezing behavior induced by caffeine in zebrafish (Figure 4(b), CTRL = 0/CAF = 11.06 ± 0.72 vs. TPH + CAF = 3.74 ± 0.82). Values of erratic swimmer events also were decreased in the animal cotreated with tocopherol and caffeine when compared with the caffeine-treated group (Figure 4(c), CTRL = 1/CAF = 4 ± 0.35 vs. TPH + CAF = 3.5 ± 0.82; *F* = 17.09; *p* < 0.05). As observed, increased latency to top observed in zebrafish treated with caffeine was prevented by alpha-tocopherol treatment (Figure 4(d), CTRL = 13.5 ± 3.75 s/CAF = 114 ± 30.9 s vs. TPH + CAF = 10.6 ± 2.32; *F* = 5.792; *p* < 0.05).

### 3.3. Oxidative Stress in Zebrafish Brain Induced by Caffeine

**Figure 5:**
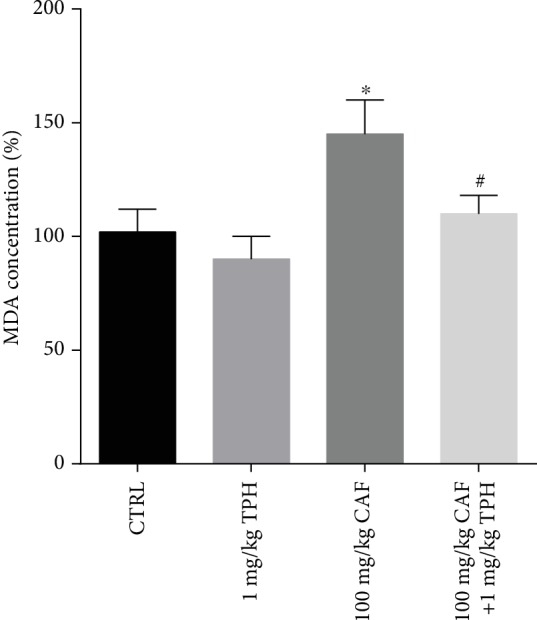
Lipid peroxidation in the brain of zebrafish treated with caffeine or caffeine and alpha-tocopherol. Data are expressed as percent of control and compared using the ANOVA-one way test followed by the Tukey test. ^∗^*p* < 0.01 vs. control.

Caffeine treatment induced significant elevation in the MDA levels in the zebrafish brain after 30 minutes of exposure (Figure 5: CTRL = 100 ± 8% vs. CAF = 154 ± 20%). As observed in Figure 3, treatment with TPH prevented MDA elevation induced by caffeine treatment in the zebrafish brain (CAF = 154 ± 20% vs. TPH + CAF = 113 ± 15%). TPH treatment did not have a significant effect on the MDA levels of the control group.

## 4. Discussion

Anxiety induced by intense caffeine intake has been widely investigated in humans [22], rodents [23–28], and fish [16, 17, 20, 29]. In the current study, we demonstrated that high doses of caffeine induces anxiety-like behavior in zebrafish, and this effect was evidenced in different behavior parameters such as thigmotaxis, freezing frequency, and erratic swimming. Maximino et al. [17] demonstrated that caffeine stimulation of A1 adenosine receptors evokes anxiety-like behavior in zebrafish. Similar results were also observed in rodents, other fish species [30], and zebrafish larvae [31]. Although caffeine effect on the CNS is not fully understood, there are some evidences indicating that a high dose of caffeine stimulates A1 adenosine receptors and generates oxidative stress in the brain [17, 32]. The association between oxidative stress and anxiety was already demonstrated in the literature [11]. Maximino et al. [33] showed that ROS production and serotonin oxidation mediate anxiety-like behavior in zebrafish. In addition, Puty et al. [34] described that antioxidant treatment prevents serotonin oxidation and anxiety-like behavior induced by mercury exposure in zebrafish. To our knowledge, this is the first study showing that alpha-tocopherol treatment prevents anxiety-like behavior induced by caffeine treatment.

Previous studies already proved the prooxidant effect of caffeine caused by inhibition of antioxidant enzymes such as catalase, superoxide dismutase, and glutathione peroxidase [35]. Despite these findings, there are few evidences showing that oxidative stress mediates behavioral changes induced by caffeine. In this current study, we demonstrated that caffeine induces lipid peroxidation in the zebrafish brain as well as that alpha-tocopherol treatment prevents this biochemical alteration. Here, we demonstrated for the first time the effectiveness of a classical antioxidant against oxidative stress and anxiety-like behavior induced by high doses of caffeine.

Due to the fat-soluble property of the alpha-tocopherol, this isomer form of vitamin E easily crosses the blood-brain barrier and can promote neuroprotective effects [11]. Previous experimental studies demonstrated that depletion of alpha-tocopherol content induces anxiety-like behavior in rats [13]. Although the anxiolytic effect of alpha-tocopherol has been previously described, it remained unclear the action of the given antioxidant against behavior changes induced by xenobiotics such as caffeine. The present study showed that alpha-tocopherol treatment was able to avoid the anxiogenic behavior elicited by caffeine treatment in zebrafish. The protective effect exerted by alpha-tocopherol was obtained using an experimental dose within range used for human treatment [36]. In addition, anxiolytic effect of alpha-tocopherol was observed in all behavior parameters analyzed in the present study. It supports our hypothesis that oxidative stress mediates the anxiety-like behavior induced by caffeine. In fact, recent studies have showed positive effect of alpha-tocopherol treatment on other neuropsychiatric diseases such as depression [37, 38]. Posterior studies should be performed to clarify the cellular and molecular mechanisms assuring the protective effect exerted by alpha-tocopherol against anxiety-like behavior evoked by caffeine. However, the current work proposes, using preclinical evaluation, that alpha-tocopherol treatment represents a promising strategy to prevent the anxiety behavior evoked by high caffeine consumption.

## 5. Conclusion

In conclusion, our data showed that alpha-tocopherol exerts a protective effect against brain oxidative stress and anxiety-like behavior induced by caffeine in zebrafish. These results strongly suggest that generation of oxidative stress in the brain mediates the anxiety-like behavior elicited by high caffeine intake.

## Data Availability

The data used to support the findings of this study are available from the corresponding author upon request.

## Supplementary Materials

Supplementary Materials The effect of alpha-tocopherol on the zebrafish anxiety-like behavior was available in Supplementary Materials.
