# Supplementary material for: Oxidative Stress Mediates Anxiety-Like Behavior Induced by High Caffeine Intake in Zebrafish: Protective Effect of Alpha-Tocopherol
Source: Oxid Med Cell Longev. 2019 Oct 21;2019:8419810. doi: 10.1155/2019/8419810 (PMC6854957; doi:10.1155/2019/8419810)
Supplement: Supplementary Materials — The effect of alpha-tocopherol on the zebrafish anxiety-like behavior was available in Supplementary Materials. [file 8419810.f1.pdf]

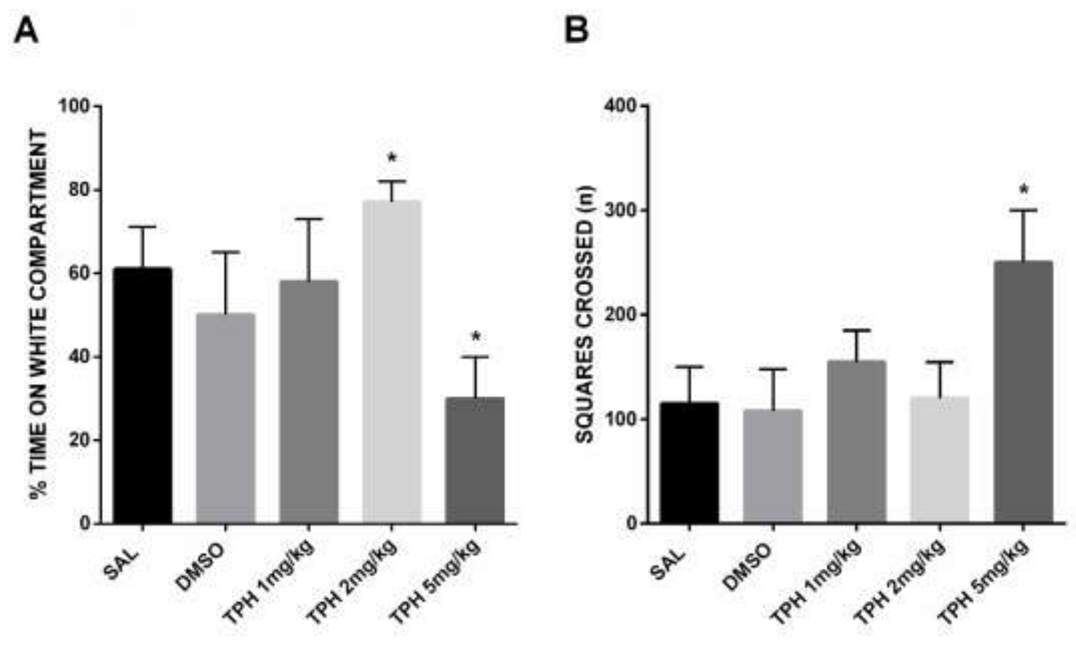

Figure 1. Effect of alpha-tocopherol (TPH) treatment on the zebrafish behavior. (A) Time spent in the white compartment and (B) motor activity in zebrafish treated with different doses of TPH. Data were expressed as media and standard error (ANOVA-one way followed by Tukey test, $^{*}p \leq 0{,}05$)
